# Supplementary material for: MicroRNAs Regulate Ca2+ Homeostasis in Murine Embryonic Stem Cells
Source: Cells. 2023 Jul 28;12(15):1957. doi: 10.3390/cells12151957 (PMC10417630; doi:10.3390/cells12151957)
Supplement: Supplementary file 1 [file cells-12-01957-s001.zip › Cells Supplemental Figures.pdf]

Figure S1

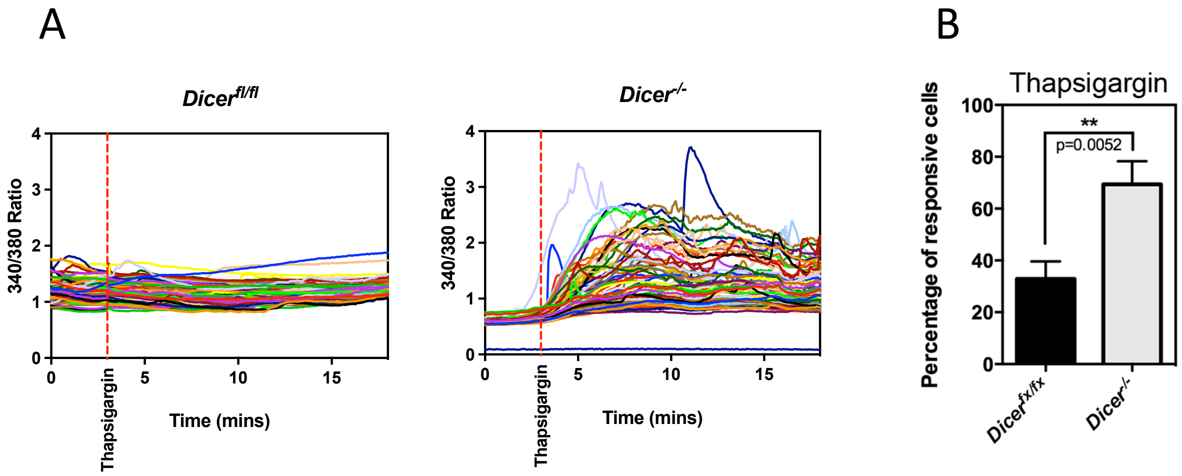

Figure S1. Cytoplasmic Ca2+ response using 200nM thapsigargin. (A) representative traces from a single experiment. **(B).** The mean level of responsive cells from six experiments. The mean value and standard deviation of the 340/380 ratios prior to thapsigargin addition were calculated for *Dicer$^{fx/fx}$* and for *Dicer$^{-/-}$* cells to give reference points and a range of basal cytoplasmic Ca^{2+} levels for each cell type. Cells that responded to thapsigargin treatment were classified as those whose 340/380 nm ratios at any time over the time course exceeded three times the standard deviation of the untreated cells.

Figure S2

A

```
miR-19 target sequence    5'- CTCGAGTCAGTTTTGCATGGATTTGCACAGAATTC -3'
                                   |||||||||||||||||||||||
                               3'- AGUCAAAACGUACCUAAACGUGU -5'     miR-19

miR-20 target sequence    5'- CTCGAGCTACCTGCACTGTAAGCACTTTAGAATTC -3'
                                   ||||||||||| |||||||||||
                               3'- GAUGGACGUGAUAUUCGUGAAAU -5'     miR-20

miR-92 target sequence    5'- CTCGAGCAGGCCGGGAGAAGTGCAATAGAATTC -3'
                                   |||||||||| ||||||||||
                               3'- GUCCGGCCCUGUUCACGUUAU -5'       miR-92

miR-96 target sequence    5'- CTCGAGAGCAAAAATGTCGTAGTGCCAAAGAATTC -3'
                                   |||||||||||   ||||||||||
                               3'- UCGUUUUUACACGAUCACGGUUU -5'     miR-96

miR-130 target sequence   5'- CTCGAGATGCCCTTTTCTCATTGCACTGGAATTC -3'
                                   ||||||||||   ||||||||||
                               3'- UACGGGAAAAUUGUAACGUGAC -5'      miR-130
```

B

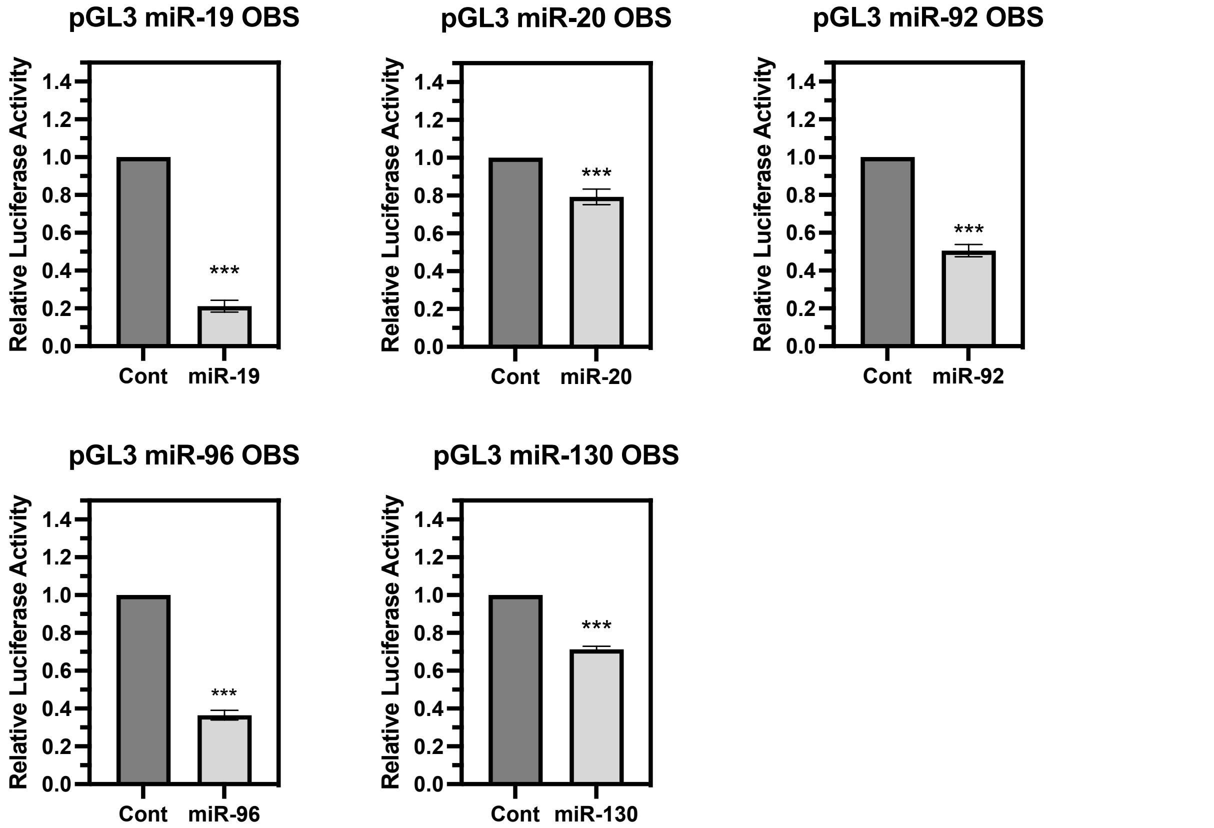

Figure S2. miRNA mimics positive controls. (A) The indicated miRNA target sequences with nearly exact matches to the specific miRNA were cloned into the 3' UTR of the luciferase expression vector. **(B)** These were tested for miRNA suppression by co-transfection into HEK 293T cells with the corresponding miRNA mimic. Values are presented relative to the effect of a negative control mimic on the indicated reporter. miR-19 $p = 0.0005$, miR-20 $p = 0.0010$, miR-92 $p = 0.0005$, miR-96 $p = 0.0005$, miR-130 $p = 0.0005$. Data are from 12 independent experiments.

Figure S3

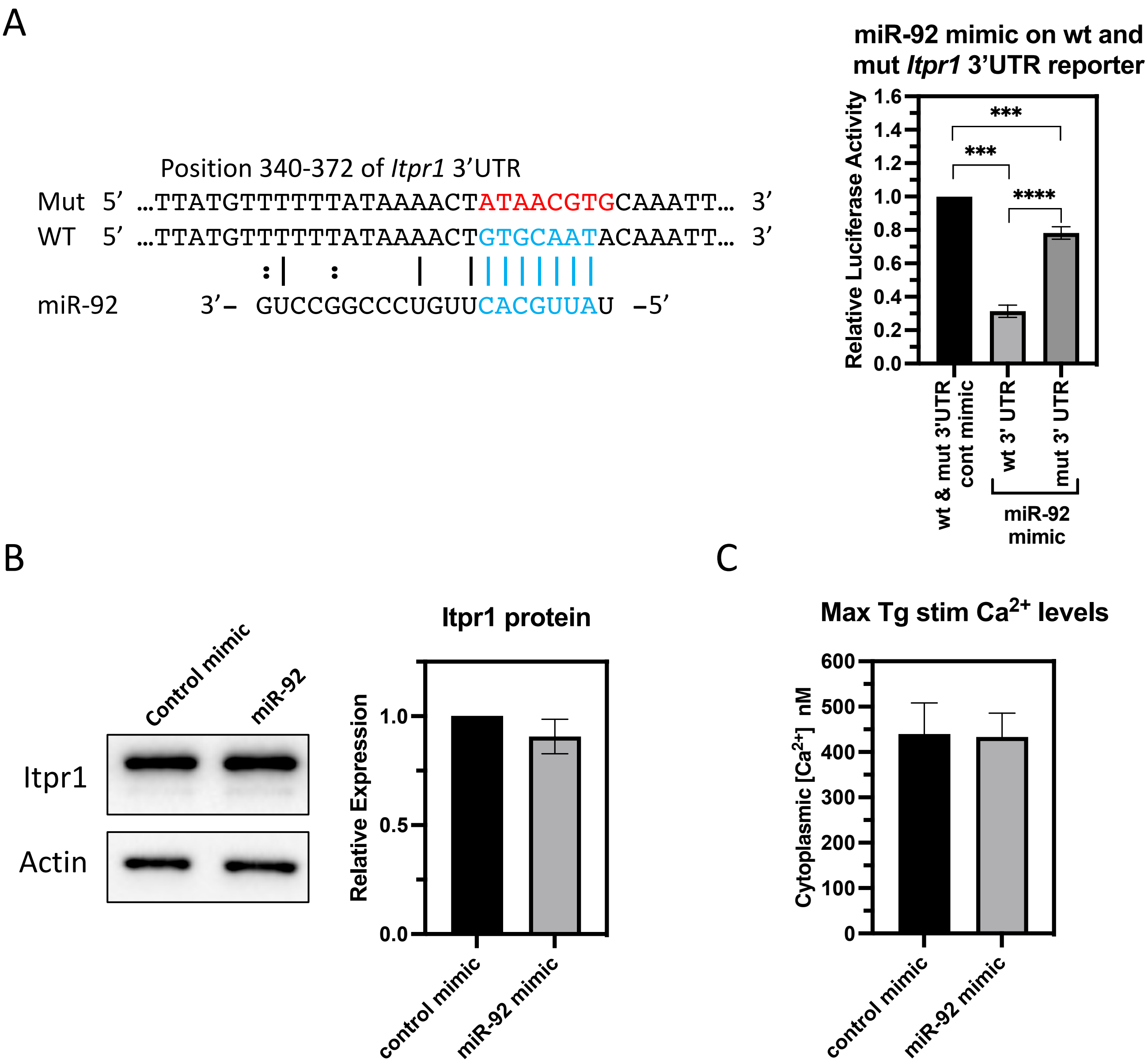

Figure S3. miR-92 effects on *Itpr1* 3' UTR reporter and endogenous *Itpr1*. (A) miR-92 regulation of the *Itpr1* 3' UTR reporter is largely dependent on its predicted target site. On the left is the sequence of the region of the *Itpr1* 3' UTR containing the predicted miR-92 target site, its ability to base pair with the seed sequence of miR-92, and the sequence of the mutated reporter to test the effect of miR-92 on the *Itpr1* 3' UTR reporter. On the right shows the results from co-transfection experiments of HEK 293T cells with the indicated reporters and the control or miR-92 mimic. Values are presented relative the effect of the negative control miRNA mimic. (wt reporter $p = 0.0005$, mut reporter $p = 0.0005$. mut versus wt reporter $p < 0.0001$). Data are from 12 independent experiments. **(B)** miR-92 has no effect on expression of the endogenous *Itpr1*. On the left is an Itpr1 Western blot of extracts from ESCs transfected with the control or miR-92 mimic. The bar chart shows a summary of the quantification results from three independent experiments. **(C)** miR-92 also has no effect on the Ca^{2+} sensitivity to thapsigargin of *Dicer*$^{-/-}$ ESCs. Maximum thapsigargin stimulated cytoplasmic Ca^{2+} levels were derived as done in Fig. 2. Data are from six independent experiments.
